# Development of Severe Hyponatremia due to Salt-Losing Nephropathy after Esophagectomy for Esophageal Cancer

**DOI:** 10.1155/2009/241283

**Published:** 2009-10-28

**Authors:** Katsunobu Yoshioka, Minako Nishio, Soichi Sano, Katsunobu Sakurai, Keiko Yamagami, Yoshito Yamashita

**Affiliations:** ^1^Department of Internal Medicine, Osaka City General Hospital, Osaka 534-0021, Japan; ^2^The Department of Gastroenterological Surgery, Osaka City General Hospital, Osaka 534-0021, Japan

## Abstract

A 72-year-old woman was admitted to our hospital for esophagectomy for esophageal cancer. On the third postoperative day, she developed polyuria (3.8 L/day), massive natriuresis, hyponatremia (112 mEq/L), hyperkalemia (5.6 mEq/L), and decreased central venous pressure, which was refractory to isotonic saline infusion. Laboratory findings indicated proximal tubular injury (high urinary *β*2-microglobulin, coexistence of hypouricemia) together with reduced aldosterone action at the cortical collecting duct. A diagnosis of salt-losing nephropathy was made and sodium correction was done with 3% saline and fludrocortisone. She responded well to therapy. The cause of hyponatremia was considered renal tubular dysfunction together with elevated antidiuretic hormone level. Postoperatively, it is important to look for the development of salt-losing nephropathy.

## 1. Introduction

Postoperative hyponatremia is usually attributed to the syndrome of inappropriate secretion of antidiuretic hormone (SIADH), which results from administration of excessive fluid in the presence of nonosmotic secretion of ADH [1]. In this setting, electrolyte-free water is retained in the body, and extracellular fluid volume (ECV) is expanded. On the other hand, salt-losing nephropathy (SLN) is defined as a renal loss of sodium that leads to hyponatremia and ECV loss [2]. Differentiation of SLN from SIADH is important because treatment of SLN is opposite from that of SIADH. However, differentiation of these disorders is problematic because clinical and laboratory findings are similar and evaluation of ECV status is difficult. Here, we report a case of postoperative hyponatremia attributed to SLN in which the patient's ECV status was monitored by measuring central venous pressure (CVP), hematocrit value, and water balance. Furthermore, we describe the underlying mechanisms of natriuresis in the present case.

## 2. Case Report

A 72-year-old woman was admitted to our hospital for surgery for esophageal cancer. At admission, her blood pressure was 120/70 mmHg and pulse rate was 76 beats per minute. She was 152 cm in height and weighted 62 Kg. Physical examination was unremarkable. Preoperatively, renal function was moderately decreased (creatinine, 0.95 mg/dL, blood urea nitrogen: 16.5 mg/dL, uric acid: 5.7 mg/dL, sodium: 144 mEq/L, potassium: 4.1 mEq/L, chloride 110 mEq/L, phosphorus 3.7 mg/dL). During the thoracoscopic esophagectomy, blood loss was 460 ml, and hemodynamics were stable.

**Figure 1:**
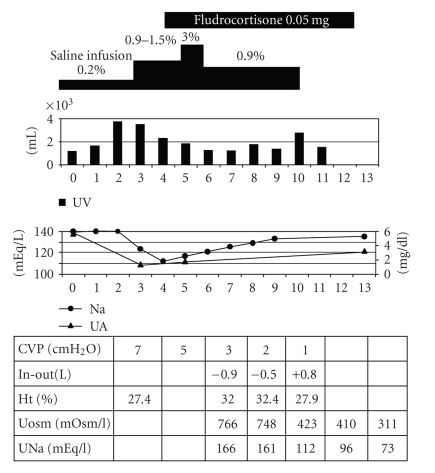
Clinical course.

Postoperatively, approximately 2 L of hypotonic fluid (0.2% saline with 4.3% dextrose) daily were administered, urine volume was stable (1.1–1.8 L), and plasma sodium levels were unchanged (140 mEq/L). However, on the third postoperative day, she suddenly developed polyuria (3.8 L/day) together with a decrease in CVP (Figure 1). Serum sodium levels dropped to 124 mEq/L on the next day. Despite volume-to-volume correction with isotonic saline, the serum sodium levels dropped to a nadir of 112 mEq/L together with hyperkalemia (5.6 mEq/L) on the fifth postoperative day, and her consciousness level deteriorated. When her urine volume was 3.8 L and 3.5 L, there was a negative water balance (without including perspiration); −0.9 L and −0.5 L, respectively. The hematocrit value increased from 27.4% to 33.7% during the 2 days without blood transfusion. The next day, our department was consulted for further evaluation. She appeared dehydrated and there was an additional decrease in her CVP to a nadir of 1 cm of H2O. Laboratory findings on consultation are shown in Table 1. Urinary sodium concentrations were markedly high and serum uric acid levels were decreased from 5.7 mg/dL to 1.4 mg/dL. Urinary N-acetyl-*β*-glucosamininase (NAG) and *β*2-microglobulin were high and the calculated maximal tubular reabsorption of phosphorus per glomerular filtration rate (TmP/GFR) was relatively low (2.4 mg/dL), suggesting proximal tubule dysfunction. Furthermore, the calculated transtubular potassium gradient (TTKG) value was low (3.6) for her serum potassium levels (4.7 mEq/L), suggesting that aldosterone action at cortical collecting duct (CCD) was impaired. These features were not consistency with the diagnosis of a SIADH. Thus, she was diagnosed as having SLN due to renal tubular dysfunction. Sodium correction was done with 3% saline and fludrocortisone was started at a dose of 0.05 mg/day. Thereafter, urinary volume decreased and there was a gradual increase in serum sodium levels to 138 mEq/L and serum uric acid levels to 3.4 mg/dL. Her consciousness level completely recovered. On the 20th postoperative day fludrocortisone was discontinued and she was discharged. She has been followed as an outpatient without recurrence of the hyponatremia.

## 3. Discussion

The present case developed hyponatremia on the fourth postoperative day together with increased urine volume, a negative balance for water, an increase in hematocrit values, and decreased CVP. Thus, ECV status was considered hypovolemic, which was not consistency with the diagnosis of a SIADH.

 The cause of hypovolemic hyponatremia was thought to be renal loss because urinary sodium concentrations were high. The cause of renal loss includes diuretic use, adrenal insufficiency, osmotic diuresis, cerebral salt wasting syndrome (CSWS), and SLN. Diuretic use, adrenal insufficiency, and osmotic diuresis were ruled out clinically and endocrinologically. CSWS was introduced in 1950 [3], when it was used to describe renal salt wasting caused by intracranial disease. Considering that the present case had no intracranial disease, it was unlikely that the cause of hyponatremia was CSWS. Some researchers claim that “SLN” does not exist. However, Maesaka presented evidence of renal salt wasting in a hyponatremic patient and recommended the elimination of “cerebral” in favor of “renal” in salt-wasting syndrome, as the absence of clinical cerebral disease would eliminate renal salt wasting from the differential diagnosis when evaluating the hyponatremic patient [4]. Thus, “SLN” does exist and the cause of hyponatremia in the present case was considered to be SLN.

 We believe that proximal tubular dysfunction existed in the present case. First, urinary concentrations of *β*2-microglobulin and NAG were high, suggesting proximal tubular injury and dysfunction. Second, hyponatremia and hypouricemia coexisted despite ECV depletion. The reabsorption of urate occurs mainly in the proximal tubule and is indirectly coupled to sodium reabsorption [5]. Patients with ECV depletion generally have hyperuricemia, because proximal tubules avidly reabsorb sodium together with urate. Therefore, the coexistence of ECV depletion and hypouricemia suggested proximal tubular dysfunction and injury. To show that reabsorption of urate is disturbed, it is essential to calculate fractional excretion of urate (FEUA). Unfortunately, urinary urate was not measured at the first account. We measured urinary urate after her serum urate increase to 3.4 mg/dL, when her FEUA was high at a level of 11.5%. This observation suggest that reabsorption of urate should have been severely disturbed when her serum urate was 1.4 mg/dL. Third, TmP/GFR was relatively low, which is characteristics of SLN and not of SIADH.

 We considered that not only proximal tubule but also another segment of renal tubules were affected, because if the proximal tubule is the only affected tubule, more distal tubules can compensate for reduced proximal tubule sodium reabsorption and severe hyponatremia rarely occurs. Final regulation of sodium resbsorption is done at the CCD. The coexistence of hyperkalemia and hyponatremia, although hyperkalemia is an unusual presentation of SLN, led us to suspect that the CCD could not compensate for reduced proximal tubule sodium reabsorption due to refractoriness to aldosterone. Under conditions of maximal aldosterone action, the TTKG should be 7–10. In the present case, however, TTKG was 3.6, suggesting aldosterone action was suppressed.

 Because final regulation of free water is done at the medullary collecting duct via the action of ADH, sodium loss itself does not necessarily cause hyponatremia. The final cause of hyponatremia was considered elevated ADH level for her plasma osmolality together with increased urinary sodium loss and intake of free water due to infusion of hypotonic fluid. Causes of nonosmotic secretion of ADH in the present case include ECV depletion and postoperative stress. To suppress ADH secretion, volume load was done with 3% saline infusion, and fludrocrotisone, which acts directly on the CCD, was administrated. The patient responded well to this treatment.

 Postoperative hyponatremia occurs despite near-isotonic saline infusion in which hyponatremia is caused by generation of electrolyte-free water during excretion of hypertonic urine; this is referred to as desalination phenomenon [1]. Although the precise mechanism underlying this phenomenon in not elucidated, the most important factor is the amount of saline that was infused to maintain blood pressure during anesthesia. Thus, it is possible that we were just observing desalination phenomenon. However, in reported cases of desalination phenomenon, hyponatremia occurred within 24 hours after surgery and the water balance was positive. So, this mechanism was unlikely in the present case. We therefore considered that a natriuresis factor other than desalination phenomenon existed in the present case.

 The driving force for proximal tubule Na reabsorption depends on electrochemical gradients generated by Na/K ATPase at the basolateral membrane. Endothelial derived inflammatory cytokines, mediated by nitric oxide production, reduce proximal tubule Na reabsorption by downregulation of Na/K ATPase activity [6]. In some pathological conditions such as systemic-onset juvenile arthritis [7] and Adult still's disease [8], systemic inflammatory responses are associated with abnormal Na transport which is involved in the pathogenesis of SLN [9]. Furthermore, reduction in Na transport due to inflammatory cytokines is refractory to aldosterone because the epithelial sodium channel at the apical membrane is also downregulated [9]. Major surgery induces a severe inflammatory response that may be related to the development of severe postoperative complications [10]. In the present case serum levels of interleukin 6 (IL-6) were elevated (34.8 pg/mL). Thus, postoperative inflammatory cytokines may be one mechanism for reduced proximal tubule Na reabsorption. However, the elevation of IL-6 was mild and other mechanisms such as pressure natriuresis due to large adrenergic surge may be involved in the mechanisms. Adrenergic surge may cause renal vasodilation and increase GFR. As a result, serum creatinine could be decreased despite dehydration.

 In summary, we encountered a patient who developed severe hyponatremia after esophagectomy for esophageal cancer. The cause of hyponatremia was considered renal tubular dysfunction together with elevated ADH level. Postoperatively, it is important to look for the development of SLN for proper management.

## Figures and Tables

**Table 1:** Laboratory data on consultation.

Urinalysis		Biochemistry		Endocrinolgy	
Osmolality	766 mOsm/l	Osmolality	251 mOsm/l	Cortisol	30.7 *μ*g/dL (4–23.3)
Sodium	166 mEq/L	Sodium	119 mEq/L	ACTH	29 pg/ml (7–56)
Potassium	52 mEq/L	Potassium	4.7 mEq/L	Aldosterone	161 pg/ml
Chloride	183 mEq/L	Chloride	88 mEq/L	PRA	1.3 ng/ml/h
*β*2-microglobulin	9.3 mg/l	Calcium	8.0 mg/dL	AVP	9.96 pg/ml
	(<0.17)	Phosphorus	3.2 mg/dL	freeT4	1.7 ng/ml (0.9–1.7)
NAG	41.0 IU/l (0.7–11.2)	Creatinine	0.5 mg/dL	TSH	1.22 *μ*IU/ml (0.5–5)
TTKG	3.6	BUN	17.8 mg/dL	Cytokines	
TmP/GFR	2.4 mg/dL	Uric acid	1.4 mg/dL	IL- 6	34.8 pg/ml (<0.4)
		Glucose	187 mg/dL	TNF-*α*	<5 pg/ml (<0.5)

NAG: N-acetyl-*β*-glucosamininase, TTKG: transtubular potassium gradient, TmP/GFR: maximal tubular reabsorption of phosphorus per glomerular filtration rate, BUN: blood urea nitrogen, ACTH: adrenocorticotropic hormone, PRA: plasma renin activity, AVP: arginine vasopressin, T4: thyroxine, T3: triiodothyroxine, TSH: thyroid stimulating hormone, IL-6: Interleukin-6, TNF-*α*: tumor necrosis factor-*α*.
